# Genes, Drugs, and Personalized Medicine—The DNA of a Pharmacogenomics Curriculum

**DOI:** 10.1007/s40670-025-02449-x

**Published:** 2025-07-14

**Authors:** Linda F. Chang, Radhika Sreedhar

**Affiliations:** 1 University of Illinois Chicago-Rockford College of Medicine, 1601 Parkview Ave Rockford, Chicago, IL 61107 USA; 2 University of Illinois Chicago College of Medicine, 840 S. Wood Street Clinical Sciences North, Ste 440, MC 718, Chicago, IL 60612 USA

**Keywords:** Pharmacogenomics elective curriculum

## Abstract

Precision medicine tailors treatment based on individual genetic, environmental, and lifestyle differences. A key component is pharmacogenomics, which informs drug response and guides personalized care. We developed a pharmacogenomics curriculum for healthcare students using constructivist principles and mastery learning assessment. Activities included case-based quizzes, real-world simulations, and team projects. Among 87 students completing the elective, average quiz scores rose from 42 to 90%, and students scored ≥ 85% on performance tasks. All reported improved ability to apply genomic data to patient care. Our model aligns with genomic EPAs and prepares students for clinical integration.

## Background

Precision medicine is an innovative disease treatment and prevention approach that considers individual variability in genes, environment, and lifestyle. A critical component of this approach is pharmacogenomics, the study of how genetic differences influence a person’s response to drugs. Pharmacogenomics drug information, pharmacokinetics, and pharmacodynamics serve as an additional tool to tailor medical treatments to a patient’s genetic profile, optimizing therapeutic outcomes while minimizing adverse effects. Evidence-based guidelines for drug-gene pairs are available to support personalized medicine [1–3]. While the field is rapidly evolving and improving with emerging data, studies have shown that many physicians feel unprepared to interpret pharmacogenomic data and lack confidence in ordering genomic tests [4, 5]. A survey of future healthcare providers found that learners valued learning about pharmacogenomics [6]. Genomic medicine education is increasingly recognized as an essential skill for non-geneticists. The Association of Professors of Human and Medical Genetics (APHMG) included pharmacogenomics in updating the core competencies for undergraduate medical education in genetics and genomics to ensure that future healthcare providers are competent in interpreting genetic information and applying it to clinical decision-making [7].

Despite its clinical relevance and growing evidence to support the clinical utility of such pharmacogenomics information, our curriculum literature review revealed a gap in this domain. Studies found that only half of pharmacy schools offer a stand-alone pharmacogenomics course, and many of the programs include educational interventions that integrate clinical cases in their curricula [8, 9]. Another study assessed the depth and extent of pharmacogenomics education in the US medical curricula and found that less than 20% of schools include some pharmacogenomics instruction, but no stand-alone or longitudinal course [10]. At the time of this literature review, our College of Medicine curriculum did not include any pharmacogenomics teaching. To address this curricular gap, we propose the integration of a pharmacogenomics course within our longitudinal pharmacology curriculum.

Constructivist theory emphasizes learning in context and creating a learning environment that is adaptive. It is a learner-centered approach where instructors provide a social environment for interactive learning, facilitating and guiding students through the learning process [11]. Our approach applied constructivist and backwards design principles to develop learning assignments that simulate the real-world work done in the healthcare industry. This student-centered course is designed to teach critical divergent thinking and active learning — not just “knowing,” but also “doing.” This involved team projects with hands-on application activities in the domains of discovery, preparation, analysis, processing, and explaining the medical information to patients.

Our assessments follow mastery learning principles to ensure that students progress to new topics only after demonstrating mastery of the current week’s material [12]. Weekly formative assessments include quizzes and team projects. The minimum mastery standard is set at 90% for quizzes and 80% for team projects. Feedback is provided and students must meet these standards before passing each week.

We present a curriculum that aims to equip healthcare students with foundational knowledge and practical skills in pharmacology through pharmacogenomics, fostering their ability to integrate genomic insights into patient care effectively. The overarching intent is to share content that allows institutions to adapt and incorporate it into various curriculum models.

### Activity

Guided by the constructivist principles and backward design, we built a three-component spiraling pharmacogenomics course to prepare students to manage complex patients in evolving healthcare settings. The first component is in the preclinical curriculum; genomic concepts were introduced in the pharmacology foundational unit, with content progressively expanding in subsequent organ systems (Table 1). Application-based learning was reinforced through short case studies integrated into the cardiovascular and neurology disease modules. In the second component, students in their clinical years engaged in complex case-solving activities incorporating genomic concepts through learning strategies in the family medicine clerkship. Although only 1 h was allocated for this topic within the clerkship, we integrated week 2 themes into the discussion session. The focus was on the impact of genetically mediated liver enzyme phenotypes on the pharmacokinetics, analgesic efficacy, and toxicity of codeine and morphine.

**Table 1:** Theme/content, learning objectives, assessment, time

Pharmacogenomics Theme/content	Learning objectives	Assessment
1. Pharmacogenomics Introduction – Nuts and Bolts (Independent content study, Virtual faculty expert panel discussion, Video patient education, Patient case – practical application) (Time: 1-h synchronous virtual meeting Student teamwork time: 5 h)	- Explain how nucleotide changes and chromosomal abnormalities can lead to altered drug metabolism - Apply your knowledge of how genetic variants affect drug absorption, distribution, metabolism, and excretion of drugs - Navigate evidenced-based pharmacogenomics databases containing information on human genetic variation and evidenced pharmacogenomics intervention -Practice patient communication and counseling skills	Pre and post quiz Weekly discussion participation Weekly hands-on activity Reflection
2. Clinical Utility of Pharmacogenomics in Neuropharmacology and Cancer Care (Independent content study, Virtual faculty expert panel discussion, Patient case – practical application Design a pharmacogenomics testing protocol for a clinic) (Time: 1-h synchronous virtual meeting Student teamwork time: 5 h)	- Design a treatment regimen taking into account the impact of different genetically mediated liver enzyme phenotypes on codeine and morphine PK (pharmacokinetics), analgesic efficacy, and toxicity – Analyze the impact of CYP2C9*2 and CYP2C9*3 alleles on the metabolism, PK, and dosage requirements of phenytoin and other anticonvulsants - Choose between two antidepressants based on the impact of genetic polymorphisms on treatment outcomes and risk of adverse outcomes - Interpret pharmacogenomics test results and apply this knowledge to make informed decisions about drug therapy in oncology	Pre and post quiz Weekly discussion participation Weekly hands-on activity Reflection
3. Clinical Utility of Pharmacogenomics in cardiovascular pharmacotherapy and Drug Hypersensitivity reactions (Independent content study, Virtual faculty expert panel discussion, Create a Dosing Algorithm for your drug-gene of choice, Pharmacogenomics and medication pharmacodynamic analysis) (Time: 1-h synchronous virtual meeting Student team work time: 5 h)	-Develop a therapeutic plan (antiplatelet, lipid management, hypertension) for a given patient based on genetic profile and currently available evidence -Analyze the impact of CYP2C9 and vitamin K epoxide reductase in warfarin response in various patient ethnic groups -Apply your knowledge of gene‐drug pairs that are associated with hypersensitivity reactions in patient case scenarios -Design treatment plan that can minimize drug hypersensitivity reactions while maximizing efficacy	Pre and post quiz Weekly discussion participation Weekly hands-on activity Reflection
4. Pharmacogenomics: Bridging Science, Ethics, and Society and critical appraisal of genomic study (Independent content study, Virtual faculty expert panel discussion, Video debate—Pharmacogenomics and Social Aspects of Patient Care, Critical appraisal write-up (Time: 1-h synchronous virtual meeting Student team work time: 5 h)	-Describe common study designs used in assessing pharmacogenomic therapy -Explain the limitations of conventional *P* < 0.05 value in a genome-wide approach study -Identify challenges associated with conducting prospective pharmacogenomics-based randomized controlled trials -Explain how pharmacogenomics may influence social and legal aspect of patient care -Identify potential challenges in maintaining patient confidentiality when using genetic data -Assess the implications of pharmacogenomic results for family members and the ethical dilemmas in disclosing hereditary risks -Explore ethical considerations in balancing access to pharmacogenomic testing with cost and healthcare resource allocation	Pre and post quiz Weekly discussion participation Weekly hands-on activity Reflection

The third component is a concentrated month-long elective for our senior medical students. Activities fostered core skills such as clinical reasoning, communication, and patient care skills. Samples of activities include (1) recording patient counseling videos on the pros and cons of genomic testing; (2) engaging in team debates on the ethical implications of genomic testing; (3) designing a genomic clinical service model; and (4) developing treatment strategies for complex patient cases that integrate genomic data, patient-centered information, and disease management (Table 2).

**Table 2:** Activities, assignment instructions, competency rubric

Activity	Hands-on activity instructions	Competency rubric
Patient education video — a 63-year-old male with incidentally discovered infrarenal aortic aneurysm	**Review the case:** Carefully review the provided patient vignette and CYP2C19 genotyping report. **Interpret the results:** Collaboratively analyze the genotyping results and determine the patient’s predicted CYP2C19 phenotype. Use CPIC or PharmGKB database as your resource. **Discuss implications:** Discuss the clinical significance of this result for the patient. **Record a patient-doctor conversation**	1. Genomic lab result interpretation 2. Health literacy skills 3. Patient engagement 4. Team work
Patient case application — a patient with refractory depression is seeking physician guidance on genetic testing options	**Assess the patient case:** review patient history and patient request. **Assess:** direct to consumer marketing genetic testing companies **Write-up:** In 250 words or less, analyze whether 23andMe genetic testing is an appropriate option for the patient described in the vignette	1. Accuracy 2. Potential limitations 3. Clinical relevance
Design an evidence-based psychotropic pharmacogenomics testing for a local mental health clinic	**Provide** an overview of Psychotropic Pharmacogenetics preemptive testing proposal including work flowchart or diagram. **Include** the evidence Supporting Preemptive Pharmacogenetic Testing and Implementation Considerations (any logistical and ethical considerations?) **Consider** cost-effectiveness data, patient acceptance, and clinician training needs **Recommendations** and rationale for the Clinic decision board members	1. Accuracy 2. Adequate literature review and evidence appraisal 3.Clinical relevance and health clinic patient-centered recommendations and considerations 4. Adequate information for a prescription or testing orders
Multidisciplinary oncology team managing breast cancer patients — team care management for different patient cancer types	**Summarize** patient cancer types and co-morbidity. **Determine** the appropriateness of hormone therapy for the following patient cases. **Identify** additional services and genomic consultations for patients. **Provide** a follow-up and ongoing monitoring plan for the patient	1. Appropriate plan and monitoring parameters 2. Identify interpatient variability factors 3. Identify which patients may benefit from hormone therapy and provide a rationale
Create a dosing algorithm for your drug-gene pair of choice	**Use** the Clinical Pharmacogenetics Implementation Consortium (CPIC) database to create a dosing algorithm for your chosen drug. **Justify** team algorithm by explaining how genetic data informs dose adjustments. **Provide** cost-effectiveness data to support or not supportive of preemptive genetic testing	1. Provide alternative effective interventions 2. List the potential harm of preemptive genetic testing 3. Support recommendations with evidence from clinical studies, guidelines, or economic analyses
Pharmacogenomics and medication pharmacodynamic analysis— a patient with history of HIV and asthma presents with yellowing of his eyes	**Review** patient case. **Identify** patient’s genomic data and medical conditions. **Assess how** genomic data, combined with pharmacodynamic principles, impacts patient care and treatment outcomes	1. Accuracy 2. Integration of dynamic principles and patient genomic data appropriately

In the concentrated, month-long elective course, students engaged in various activities to ensure their understanding of core concepts. They completed a baseline quiz (two sample questions from each weekly theme), assigned readings, real-world pharmacology simulations, weekly virtual Zoom discussions, weekly quizzes (20 items related to the weekly theme), and reflective exercises. Students’ assignments were team-based projects with hands-on activities focused on discovering, analyzing, and translating data/medical information into patient care. They engaged in team-based activities to interpret and design cost-effective pharmacogenomic testing programs and worked collaboratively to create patient-focused videos that explained the role of pharmacogenomic testing. These activities ensured students navigated and applied information from reliable pharmacogenomics resources, such as the Clinical Pharmacogenetics Implementation Consortium CPIC and PharmGKB, while highlighting pharmacokinetic and pharmacodynamic principles. A structured debate allowed students to explore the social and legal implications of pharmacogenomics in patient care, fostering critical thinking and ethical reasoning. Weekly virtual Zoom discussions with experts in various clinical settings were held on pharmacogenomics-related clinical integrations and challenges.

To evaluate the course’s effectiveness, a mixed-methods approach was employed using quantitative and narrative data. Quantitative data were collected through weekly case-based quizzes to assess foundational knowledge, as well as performance on patient care application exercises and video simulation activities to evaluate skills. Additionally, the course followed a mastery learning approach, with a minimum proficiency level (MPL) set at above 90% for the weekly quiz and above 80% for the team activities. Students are required to retake the quiz and redo their team activities until they meet the minimum proficiency level (MPL). They are given multiple opportunities to successfully achieve the set MPL. Narrative data were gathered through students’ self-reflection assignments, which captured their attitudes toward pharmacogenomics and how they envisioned applying the knowledge in their future clinical practice. This data was captured at the end of each week.

## Results

A total of 87 students completed the concentrated month-long elective curriculum. *Knowledge*: The average quiz score improved from 42% on the pre-quiz to above 90% (MPL-90%) on the post-quiz. *Skills*: Students achieved an average score of at least 85% on the EPA-aligned rubric for grading videos, case-based applications, and student debate tasks (MPL-80%). *Attitudes*: All students agreed or strongly agreed that their knowledge and ability to apply patient genomic data to patient care improved due to this course. Students’ constructive feedback included a request for more detailed assignment rubrics and clearer expectations for the first 2 weeks of the course. Additionally, they suggested emphasizing to future students the importance of beginning weekly activities on day 1, rather than completing all tasks in a single day. In addition, students also provided feedback on the weekly reflection assignments. Table 3 highlights a summary of common themes from students’ weekly narrative reflection. However, no further qualitative analysis was performed.

**Table 3:** Summary of student reflections

Core concepts	Themes	Comments
Pharmacogenomics foundations	Higher confidence in navigating genomic evidence-based databases and using genomic data along with drug principle data to take care of patient conditions	“mostly surprised by how much drug kinetics/genomics details available” “so much potential ways for drug-drug interactions” “by participating in the weekly activities and discussions, I understand how to manage patients effectively”
Communication	The video patient education assignment highlighted the challenges students faced in maintaining health literacy, particularly in avoiding medical jargon and personalizing patient education about the pros and cons of genomic information, along with its ethical concerns	“most difficult part is to make information palatable to what a real patient needs” “most challenge is on properly translating complex a drug and patient pharmacogenomics information into language understood by the patient” “difficult to balance how much information to give patient without overwhelming them”
Concepts integration	Students gained insights into the differences between clinical significant side effects, drug-gene or drug-drug interactions, and treatment strategies for given conditions. They also valued the importance of basic science integration into clinical patient care	“any drug can have drug-drug interactions, also with genomic variation. Some drugs have more volatile and dangerous than others” “interpretation of pharmacogenomics literature involves clinical, statistical, and basic science skill sets. All 3 are necessary to critically appraise and use these data for patient care” “continue critically thinking throughout the course on how to implement genomics data in everyday clinical life”
Teamwork	Students quickly formed team dynamics and identified a team working schedule effectively	“I think as the course went on, we became better at working with each other and more efficient” “my team is excellent, we all contributed to the work early on” “I think it’s good to know there are genomic experts we can count on in the clinical setting”

## Discussion

The National Human Genome Research Institute developed genomic Entrustable Professional Activities (EPAs) encompassing areas from diagnostic testing to treatment interventions [13]. We demonstrate the successful implementation of a pharmacogenomics curriculum in medical school to address EPAs, particularly EPA 4 (Enter and discuss orders and prescriptions) and EPA 7 (Form clinical questions and retrieve evidence to advance patient care). This success was demonstrated by students’ enhanced knowledge scores and achievement of the minimum proficiency level for activity outcome assignments. This curriculum reflects the core concepts of constructivist learning theory by emphasizing active knowledge construction, contextual learning, and learner engagement. Foundational content and case-based discussions in the preclinical years laid the groundwork, while an hour-long discussion session on clinical cases in clerkships allowed students to apply what they had learned to identify pharmacogenomics resources and discuss their understanding in practice-based settings. The elective course for senior students provided a deeper, self-directed learning opportunity, characteristic of constructivist environments where learners build on prior knowledge and interests. Although the month-long elective online format provided flexibility and accessibility across campuses, it limited real-time peer collaboration—an important element of (in-person) social constructivism. However, the flexible, integrated model supports virtual team learning and application across diverse clinical contexts, and the opportunity for students to consult weekly with content faculty expert clinicians further promotes the meaningful construction of knowledge aligned with real-world clinical competencies.

Our curriculum model includes a comprehensive proposal that is easily adaptable by other institutions, whether through brief standalone sessions or small-dose integration into existing curricular structures. At our institution, opportunities to introduce pharmacogenomics were incorporated into in-person sessions during the preclinical years and with a focus on week 1 content (Table 1), as well as in-person discussions during the clinical clerkship. The concentrated, month-long elective course was delivered in a virtual format, offering flexibility while maintaining depth of content.

There are limitations in our study. One limitation of this study is that the concentrated month-long elective course was optional for senior students, and the results primarily reflect the performance and engagement of students who were already interested in the subject matter. As such, the findings may not be generalizable to the broader student population. Additionally, the course followed a mastery learning approach, with a minimum proficiency level (MPL) set at above 90% for the weekly quiz and above 80% for the team activities. Students were given multiple opportunities to achieve this threshold, which not only may have contributed to the overall high performance but also limit comparability to traditional assessment models. The majority of the students passed the quizzes and team activities under two attempts. In addition, the narrative feedback component is that the attitudes data were descriptive and based on open-ended reflections rather than a validated assessment tool, which may affect the reliability and comparability of these findings. Another core limitation is a lack of direct patient engagement within the course that resulted in an inability to capture real-world patient outcomes, and we were not able to follow students longitudinally to determine if they applied the skills in their practice. This highlights the need for future studies to explore integrating hybrid models that combine online learning with in-person clinical rotations or patient interactions, as well as longitudinal studies to evaluate how the skills and knowledge acquired in the course translate into practice and impact patient care over time. Future iterations will incorporate AI technology-based tools such as AI patient simulations and models to deliver precision education and enhance pharmacogenomics education.

Overall, this curriculum effectively equips healthcare students with foundational knowledge and practical skills in pharmacogenomics. The outcomes of assessment and positive feedback on reflection assignments highlight the effectiveness of this approach in preparing future healthcare providers to integrate pharmacogenomics into clinical decision-making. Given the success of this approach, we plan to integrate additional hands-on activities into the general curriculum in upcoming years.
